# Exploring Fungal Biodiversity in Crop Rotation Systems: Impact of Soil Fertility and Winter Wheat Cropping

**DOI:** 10.3390/plants14010065

**Published:** 2024-12-28

**Authors:** Srdjan Šeremešić, Sonja Tančić Živanov, Miloš Rajković, Vladimir Aćin, Stanko Milić, Brankica Babec, Snežana Jovanović

**Affiliations:** 1 Faculty of Agriculture, University of Novi Sad, Sq Dositeja Obradovica 8, 21000 Novi Sad, Serbia; srdjan.seremesic@polj.uns.ac.rs; 2 Institute of Field and Vegetable Crops, Maksima Gorkog 30, 21000 Novi Sad, Serbia; sonja.tancic@ifvcns.ns.ac.rs (S.T.Ž.); vladimir.acin@ifvcns.ns.ac.rs (V.A.); stanko.milic@ifvcns.ns.ac.rs (S.M.); brankica.babec@ifvcns.ns.ac.rs (B.B.); 3 Institute for Medicinal Plants Research “Dr. Josif Pančić”, Tadeuša Košćuška 1, 11000 Beograd, Serbia; rajkovicmilos@gmail.com; 4 Breeding Department, Maize Research Institute Zemun Polje, 11185 Belgrade, Serbia

**Keywords:** chernozem, fungi, plant–soil interactions, fungal biodiversity, crop rotation, tillage, phosphate-solubilizing, organic matter decomposition, soil fertility

## Abstract

This study investigated soil fungal biodiversity in wheat-based crop rotation systems on Chernozem soil within the Pannonian Basin, focusing on the effects of tillage, crop rotation, and soil properties. Over three years, soil samples from ten plots were analyzed, revealing significant fungal diversity with Shannon–Wiener diversity indices ranging from 1.90 in monoculture systems to 2.38 in a fertilized two-year crop rotation. Dominant fungi, including *Fusarium oxysporum*, *Penicillium* sp., and *Aspergillus* sp., showed distinct preferences for soil conditions such as pH and organic matter (OM). Conservation tillage significantly enhanced fungal diversity and richness, with the highest diversity observed in a three-year crop rotation system incorporating cover crops, which achieved an average winter wheat yield of 7.0 t ha^−1^—47% higher than unfertilized monoculture systems. Increased OM and nitrogen levels in these systems correlated with greater fungal abundance and diversity. Canonical correspondence analysis revealed strong relationships between fungal communities and soil properties, particularly pH and calcium carbonate content. These findings highlight the importance of tailored crop rotation and tillage strategies to improve soil health, enhance microbial biodiversity, and boost agricultural sustainability in temperate climates, providing valuable insights for mitigating the impacts of intensive farming and climate change.

## 1. Introduction

Over the past few decades, human activities have contributed to a significant decline in the biodiversity of plants and soils in ecosystems mostly by intensified land use and accelerated climate changes [1]. Therefore, ecological and environmental sciences are increasingly engaged in biodiversity research and its influence on ecosystem functioning [2]. It is known that soils are one of the largest biodiversity reservoirs that support the greatest diversity of organisms on the planet, and soil biodiversity is known to be a critical regulator of ecosystem function in natural ecosystems [3,4]. However, the exact influence of soil microbial biodiversity on the regulation of soil multifunctionality is not fully understood. It is known that the biochemical activity of microorganisms affects the pedogenetic process, nutrient cycling, organic matter decomposition, and the production of enzymes and acids, thus participating in the creation and maintenance of soil health. It has been confirmed that fungi play a key role in soil microbiome primarily due to their role in the decomposition of plant residues [5,6]. Consequently, fungal activity in soil leads to quantitative and qualitative changes in soil organic matter (OM) and exerts a significant relationship between fungal activity and OM preservation in agroecosystems [7]. In addition, fungi can change soil enzyme activity patterns and affect overall microbial activity, which in turn can modify soil health and quality manifestation [8,9]. Nevertheless, the mechanisms that lead to organic matter enhancement are not fully explained as many interactions could lead to different outcomes [10].

Ultimately, all these findings suggested that human intervention through cropping management that affects fungal activity could improve ecosystem functions in cultivated soils, especially those used in intensified agricultural ecosystems. In many cases, these improvements include different approaches and complex cropping adjustments, with crop rotation being a cornerstone of advanced cropping strategies [11]. In agricultural production, crop rotation has been proven to be a favorable and efficient management approach that can improve soil health and crop yields while reducing pathogens and plant soil-borne diseases in comparison to monoculture [12,13,14]. However, to test the efficiency and functionality of the specific crop rotation, investigations must be conducted in the “ambience” of long-term stationary trials [15]. Stationary multi-year experiments are an irreplaceable source of information on the state of the agroecosystem and the mutual compatibility of its elements [16]. Therefore, experimental plots comprise a vital scientific infrastructure designed to investigate the primary challenges encountered in agricultural production while offering guidelines aimed at achieving sustainability [17].

The choice of crop species, their rotation sequence, and crop diversity play a key role in shaping the abundance and composition of the soil microbial community, affecting phytopathogenic, non-pathogenic, and beneficial microorganisms. Efficient crop rotation can lead to the decline of pathogenic fungal populations due to the antagonistic activities of coexisting soil beneficial fungi and the natural mortality of pathogens that lack a plant host [18]. On the contrary, agricultural production in monoculture with a susceptible host can result in an increase in pathogens with a wide host range or pathogens that produce long-lived survival structures in soil and that are challenging for further control. In summary, crop rotation can be used as an effective management strategy to promote beneficial microbes for plant development and pest control, organic matter improvement, and soil biodiversity in general.

Primary tillage and cultural practices during the growth of preceding crops can significantly affect fungal communities in agricultural soils, with different responses observed for phytopathogenic and beneficial fungi [19]. Due to the limited research that has been conducted on the soil microbiome in conservation tillage systems on Chernozem in the Panonnian Basin, our research was focused on crop rotation effects and the comparison of conservation (CT) and common moldboard plowing tillage (PT) systems on winter wheat, which is one of the most important crops for the studied area. The Pannonian plane covers the northernmost part of Serbia, north of the Sava River and northeast of the Danube River [20]. In this region of Serbia, approximately 80% of cropland is covered with fertile Chernozem-like and meadow soils, which, combined with appropriate agricultural practices, can produce conditions for stable and high winter wheat yields. By better understanding the impact of crop rotation under contrasting soil cultivation on the soil microbiome and the interaction with cultivated plants, we can significantly contribute to adjusting the cropping technology to result in more efficient resource usage and alleviate the negative impact of climate change on agriculture.

This work highlighted the importance of crop species selection and agronomic management on microbial soil biodiversity in agricultural production systems. We assessed the impact of the cropping system on the following: (1) soil fungal diversity; (2) structure (abundance and composition) of pathogenic and non-pathogenic fungal species; (3) relationships between pathogenic, beneficial, and non-pathogenic fungi and major soil chemical parameters (CCA).

## 2. Results

### 2.1. Chemical Soil Properties

In our study (Table 1), the values of the pHKCl ranged from 7.14 to 7.45, which indicated a slightly alkaline reaction and represented a common soil pH for typical Chernozem soil in our pedo-climatic conditions. The results of pH H_2_O mirrored those for pHKCl. The investigated plot can be considered high in CaCO_3_ content because Chernozem is rich in calcium, which can affect microbiome activities. Excluding non-fertilized plots (N2P and N3P), PT showed higher values of CaCO_3_ compared with CT. The lowest values of OM were found in the unfertilized plots while higher OM was found in the MOP monoculture. The values of soil OM found at the N2P, N3P, and F2C are still considered average OM content according to national classification systems. A higher OM content, compared with the other rotation systems, was found in the WW monoculture with plowing and 3-year rotations with cover crops. There were no considerable differences in the total N content among different systems. The biggest differences in soil properties among investigated treatments were found in readily available P and K as a consequence of the long-term fertilization legacy (>50 years of experiment) and the accumulation of P and K in the soil. The highest values of readily available P and K were found in plots where cover crops were grown (F2Ccc and F2Pcc).

### 2.2. Winter Wheat Yield

Winter wheat yield and the corresponding crop residue additions represent important carbon inputs that influence fungal activity in the soil. The highest yield was obtained on the plot with the 3-year crop rotation and where the cultivation of cover crops was applied. The lowest yield of WW was determined on the unfertilized plot of a 2-year crop rotation where WW was grown in combination with maize using PT, which continuously records statistically significant lower yields compared to other cropping systems. The biggest difference between plowing and conservation tillage was found between the PT and CT monoculture, while in fertilized 2-year and 3-year rotations, the differences in CT and PT are substantially smaller. It can also be observed that long-term PT in the WW monoculture can match the 2-year WW rotation but not the 3-year WW system (maize-soybean-winter wheat). It can also be noted that some systems, especially MOC, MOP, F3P, and F3Ccc, have a higher standard deviation and variation from the average yield at the level of cca ± 3 t ha^−1^. Generally, significant differences in yield were mostly pronounced between fertilized and unfertilized systems.

### 2.3. Diversity of Soil Fungi

An analysis of the three-year-based biodiversity of ten different soil samples identified a total of 32 different fungal species grouped in 24 genera. The total number of species identified in samples MOC—F3Pcc was 17, 17, 14, 22, 19, 16, 20, 18, 20, and 21, respectively. The species that were predominant in all ten samples were as follows: *Acremonium* sp., *Fusarium oxysporum*, *Macrophomina phaseolina*, *Mortierella* sp., *Penicillium* sp., and *Pythium echinulatum*. Also, the dominant species that were found in nine out of ten soil samples were as follows: *Alternaria* sp., *Aspergillus* sp., *Fusarium solani*, *Hyalodendron*, *Pythium* sp., and *Rhizopus* sp.

The most common soil fungi identified were OM decomposers, but there were many phytopathogenic fungal species such as *Alternaria* sp., *Fusarium* spp. (eight different species), *Macrophomina phaseolina*, *Bipolaris* sp., and *Pythium* spp. Among species that are known to be beneficial for plant growth promotion or biocontrol, a few species were isolated—*Trichoderma* spp., *Gliocladium* sp., *Chaetomium* sp., *Paecilomyces* sp., and *Penicillium* spp. (Table 2).

Soil fungi with an abundance of less than 500 CFU/g soil can be considered rare species, while those with an abundance of more than 500 CFU/g soil can be considered common species. As seen in Table 2, one species can be rare in one soil sample and common in another, for example, *F. oxysporum*, *Hyalodendron*, *Trichoderma*, *Rhizopus* sp., etc.

The lowest average number of species was registered in N2P, while the highest was in N3P. The lowest average abundance was registered in MOP, while the highest was in F3Ccc. After all, Shannon–Wiener’s Diversity Index (SWDI) indicated the lowest diversity in MOC, and the highest in F2C following N3P, F3C, F3P, and F3Ccc (Table 3).

**Figure 1:**
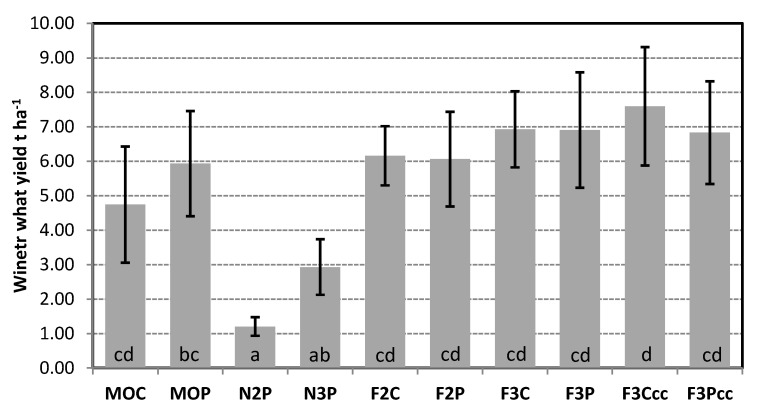
Winter wheat yield (t ha^−1^) at the experimental field (2020–2023) MOC: WW monoculture CT; MOP: WW monoculture PT; N2P: unfertilized WW PT; N3P: unfertilized WW PT; F2C: fertilized 2-year WW CT; F2P: fertilized 2-year WW PT; F3C: fertilized 3-year WW CT; F3P: fertilized 3-year WW PT; F3Ccc: fertilized 3-year WW cover crop CT; F3Pcc: fertilized 3-year WW cover crop PT. Histogram bars marked with the same letter do not differ significantly at *p* < 0.05.

The highest diversity of soil fungi in soil samples F2C, N3P, F3C, F3P, and F3Ccc can be seen in Figure 1 as well. In Figure 1, the relationship between soil fungal community and environmental factors such as physical and chemical soil properties is shown, and F3P, F2C, F3C, F3P, and F3Ccc showed up as main “centers” of the species distributions.

### 2.4. Relationship of Soil Fungi with Soil Physical and Chemical Properties

**Figure 2:**
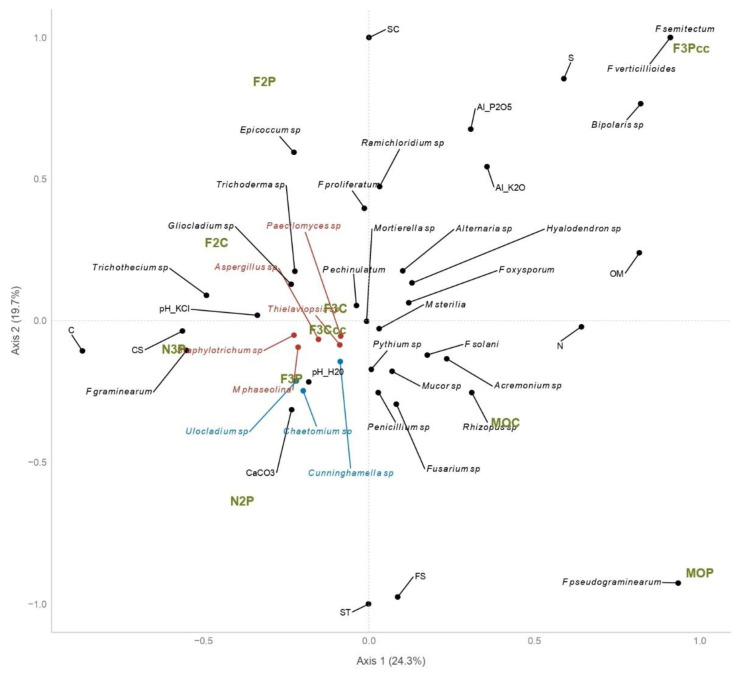
The relationship of soil fungi with physical and chemical soil properties analyzed by canonical correspondence analysis (CCA): cropping systems (MOC, MOP, N2P, N3P, F2C, F2P, F3C, F3P, F3Ccc, F3Pcc,), calcium carbonate (CaCO3), organic matter (OM), total nitrogen (N), soil reaction (pHKCl) and (pHH_2_O), available phosphorus (AL_P2O5), available potassium (AL_K2O), the percentage of coarse sand (CS), fine sand (FS), silt (S), clay (C), sand total (ST CCA), silt and clay (SC).

The relationship between the soil fungi and the soil’s physical and chemical properties was analyzed by canonical correspondence analysis (CCA), and the results showed (Figure 2) that most fungi related positively with the pH of the soil, CaCO_3_, the percentage of coarse sand (CS), and clay (C).

The soil fungi with the closest and most positive relationship to pH were *Ulocladium* sp., *Chaetomium* sp., *Chunninghamella* sp., *M. phaseolina*, *Thielaviopsis* sp., *Paecilomyces* sp., *Aspergillus* sp., and *Staphylotrichum* sp. (Cluster 1—Red color in Figure 2). The species *Ulocladium* sp, *Chaetomium* sp., and *Chunninghamella* sp. had the most positive relationship to CaCO_3_ (Cluster 2—Blue color in Figure 2) as well. *F. graminearum*, *Trichothecium* sp. and *Staphylotrichum* sp. correlated positively with coarse sand (CS). *Bipolaris* sp., *F. verticillioides* and *F. semitectum* showed preferences for soil conditions found in the F2Pcc. *Fusarium pseudograminearum* was found only in MOP.

According to Figure 2, soil samples where plowing was applied, N2P, MOP, F2P, and F3Pcc, were singled out as specific and unique samples. On the contrary, soil samples F3C, F3Ccc, and N2P-N3P were grouped according to their similarities.

## 3. Discussion

The CCA is designed to extract synthetic environmental gradients from ecological datasets. The gradients are the basis for succinctly describing and visualizing the differential habitat preferences (niches) of taxa via an ordination diagram [21]. Such a diagram (CCA) shows the patterns of variation in the community composition that can be explained best by the environmental variables and also approximately visualizes the “centers” of the species distributions along each of the environmental variables [21]. Such diagrams effectively summarized the relationships between the fungal community and environment—physical and chemical soil properties in our study.

In our study, conservation tillage generally increases fungal diversity and richness compared to conventional tillage, which was also found in some other studies [22]. The assumption is that fungal communities respond to the tillage and intensity of soil disturbance as hyphal networks, which can be disturbed by plowing and frequent soil interruption [23]. The 3-year crop rotation gave higher crop biomass and potential fresh C biomass that can be incorporated in the soil compared with other systems, and the presence of legumes as cover crops in the F3Ccc and F3Pcc may result in a more efficient conversion of residual C to OM [24,25]. This could lead to better soil moisture retention, higher microbial diversity and activity, and higher winter wheat yield. This was confirmed by our study, where soil samples with the highest average number of fungal species and SWDI as main centers of biodiversity obtained the highest WW yield (F3Ccc, F3P, and F3C). Considering the long-term cropping practices in this trial, the results obtained can be observed as a consequence of the legacy of the established soil equilibrium between the climate, crops, and crop management. Over a long period, specific cultural practices can produce cumulative effects and exert a specific impact on WW yield formation. Consequently, a considerate combination of management practices including crop rotation, fertilization, and tillage can contribute to the yield stability and sustainability of 3-year WW rotations in temperate conditions [26]. This also could be beneficial in maintaining favorable soil properties and preventing potential OM depletion. Higher OM levels and total nitrogen are associated with increased fungal richness and diversity [27]. The organic matter content significantly influences the composition and structure of soil fungal communities [28]. It is known that species such as *Mucor* sp. and *Trichoderma* spp. prefer higher amounts of OM [29]. This was confirmed by our previous research [30] and in the current study, where *Trichoderma* sp. was found in soil samples with such characteristics. *Trichoderma* species are known for their dual role in nutrient cycling and pathogen suppression. By decomposing organic matter, they release essential nutrients like carbon, nitrogen, and phosphorus into the soil. Additionally, *Trichoderma* spp. produce antifungal compounds and enzymes that inhibit the growth of soil-borne pathogens, promoting plant health and reducing the need for chemical pesticides. Soil pH-KCl analyses in our study indicated only small differences among treatments and generally showed values (>7) that do not favor fungal development. However, soil pH has a decisive influence on soil fungal diversity, with some genera adapting to a wide range of pH [31]. In some other studies, fungal diversity is unaffected and only weakly related to pH, indicating that specific communities are adapted to soil pH [32]. On the other hand, Narayana et al. [33] showed that management systems had a significant influence on soil pH and bulk density, which were positively correlated with the fungal community composition of agricultural soil located in north-central Mississippi, USA, which is similar to our results. According to CCA in our study, the closest and most positive relationship was observed between soil fungi with pH and CaCO_3_, which partly agrees with the results of Puangsombat et al. [34]. A study by Tedersoo et al. [35] revealed that soil pH explained 1.5% of the variation in the total fungal community composition in OM-rich soil.

Given this, microbial community variation is usually driven by multiple environmental factors, and cropping systems must be integrated with pedo-climatic conditions. It is already confirmed that some species such as *Paecilomyces* sp. and *Chaetomium* sp. are able to grow in the presence of Na and Ca salts [11]. Likewise, the soil P content of the investigated treatments did not significantly affect the fungi community differentiation [35]. Generally, a high soil P concentration reduces the diversity of arbuscular mycorrhizal fungi-colonizing plants, but crop species are more important in determining the community, except at the highest concentration [36]. In our study, the unfertilized plots (N2P and N3P), where a lower level of available potassium was observed, showed a shift in the fungal community composition, abundance, and diversity [37]. *Penicillium*, *Aspergillus*, *Rhizopus*, *Fusarium*, *Trichoderma*, and *Gliocladium* are commonly researched phosphate-solubilizing fungi, which are beneficial microorganisms that play a pivotal role in plant growth by increasing solubility and the effect of phosphorus in the soil by secreting organic acids that chelate phosphate ions, thereby increasing their solubility in the soil [38,39]. In our study, *Penicillium*, *Aspergillus*, *Fusarium*, *Trichoderma*, and *Gliocladium* species are mainly identified in N3P, F2C, F3C, F3P, and F3Ccc, the soil samples with the highest diversity according to SWDI 2.25, 2.38, 2.17, 2.15, and 2.11, respectively. Furthermore, phosphate-solubilizing fungi can interact with other soil microorganisms, including nitrogen-fixing bacteria and arbuscular mycorrhizal fungi that further enhance plant growth and nutrient absorption. In addition to their phosphate-solubilizing role and decomposing organic matter and enhancing carbon and nitrogen cycling in soils, *Penicillium*, *Aspergillus*, *Trichoderma*, and *Gliocladium* species are also pathogen-suppressive fungi that contribute to disease management in agricultural soils. These fungi produce secondary metabolites that inhibit pathogenic microbes or outcompete them for resources. Their presence in soil can create a microbial environment that is less conducive to disease outbreaks. Fungi like *Fusarium oxysporum* also highlight the dual nature of fungal impacts on soil health. While some strains are pathogenic, others are non-pathogenic and can promote plant growth by solubilizing phosphorus and producing phytohormones.

According to Islam et al. [40], *Mortierella* was positively associated with NO_3_, N, and Cu and negatively correlated with Cl, but its role in the inhibition of soil-borne plant pathogens remained unclear. As members of the order Mortierellales, these fungi are mainly known for their saprotrophic lifestyle as efficient decomposers of complex organic materials, including cellulose, hemicellulose, and lignin. Some *Mortierella* species produce plant growth-promoting substances such as indole-3-acetic acid (IAA) and other phytohormones, which stimulate root growth and overall plant development [41]. In our study, *Mortierella* sp. was present in all soil samples, but with its highest abundance (4.17 × 10^3^ CFU/g) in the monoculture (MOC) where a high abundance of pathogenic species was recorded as well. Furthermore, nitrate-nitrogen, copper, calcium, potassium, and chlorine were associated with a number of beneficial fungal genera but not with pathogenic fungal genera [40].

Phytopathogenic species such as *Alternaria* spp., *Aspergillus* spp., *Fusarium* spp., *Macrophomina phaseolina*, *Rhizopus* sp., *Penicillium* spp., and *Pythium* spp. are expected to be frequent in agricultural soils, especially in monocultures where the inoculum of such pathogens increases constantly, which is confirmed by our research (MOC—Figure 1). The predominant species registered in this study were *Acremonium* sp., *Alternaria* sp., *Aspergillus* spp., *Fusarium oxysporum* and *F. solani*, *Macrophomina phaseolina*, *Mortierella* sp., *Penicillium* spp., and *Pythium* spp. Which were present in 9–10 out of 10 soil samples tested in high abundances (more than 500 CFU/g). The abundance of certain *Pythium* species has been found to be correlated with various soil chemical properties, including pH, calcium and magnesium content, cation exchange capacity, and clay content [42,43]. According to Gahagan et al. [44], the abundance of *Pythium* spp. was lower in the corn–soybean–wheat rotation but higher by up to 10.6% under a wheat monoculture in Canada, which is in accordance with the results obtained in our study. Islam et al. [40] confirmed that *Fusarium* species were the predominant pathogenic fungi in Canadian agricultural field soils and their abundance was strongly impacted by the crop rotation. Their research indicated that *Fusarium* was the fungal genus most commonly associated with cereal–cereal monoculture and least common in the oilseed–pulse cropping sequences. In Serbia, small-grain cereals have been one of the most cultivated crops for centuries, and Fusarium head blight is the most common disease in these crops. Therefore, the identification of five different *Fusarium* species with high abundance in wheat monoculture (MOC and MOP) in our study is not surprising.

Furthermore, most of the species mentioned above are soil fungi that spread easily, are thermophilic and heat-resistant, and have a cosmopolitan distribution [45]. In Serbia, some of those species were found as a common fungal community in alluvium soil samples from Malo Rudare, such as *Penicillium*, *Aspergillus,* and *Cladosporium* spp. by Kiković et al. [46], but also in reservoirs of lakes and rivers, such as *Penicillium*, *Aspergillus*, *Cladosporium*, *Fusarium*, *Rhizopus*, *Mucor*, *Phoma*, and *Verticillium* sp. by Ranković [47]. Moreover, the obtained results are in accordance with the fungal diversity results from authors all over the world, where these species are reported as common species in soybean soil rhizosphere in India [48], soil and litter samples of Forest Reserve in Trat Province in Thailand [34], and litter samples from different forest types in China [10]. It is known that many taxa found in soils are similar in tropical and temperate latitudes [45].

After all, according to the average number of fungal species registered and the SWDI in our study, the lowest values were registered in monoculture (MOC)—17 species and SWDI 1.90, as expected, and N2P—14 species and SWDI 2.08, where the lowest WW yield was obtained as well. The highest SWDI was registered in F2C (2.38), following N3P (2.25), F3C (2.17), F3P (2.15), and F3Ccc (2.11), and was followed by a WW yield increase when comparing the MOC. The results of Narayana et al. [33] showed the highest SWDI in a conventional tillage–cover crop (CT-cc) system with a maize–soybean crop rotation, with different tillage practices and winter vegetative covers in north-central Mississippi. The differences in the SWDI could also be explained by the diversity in weed infestation [49], as the cropping system varied in weed phytocoenology. Previous research by Tančić Živanov et al. [30] reported the highest diversity of soil fungi in chernozem, fluvisol, and arenosol, all used as arable land or garden soils. In a previous study conducted on the same experimental plots, fertilized treatments showed a higher concentration of fungi compared with unfertilized treatments [45]. It is known that the diversity of soil microbes depends on the availability of nutrients, soil quality, and types of organic carbon inputs, such as the vegetative debris of cultivated crops, which play an important role [50].

In this study, culture-dependent methods were used for fungal isolation, growing fungi on artificial media under controlled laboratory conditions. These methods are inherently biased toward fungi that can grow in artificial environments, often underestimating the diversity of uncultivable or slow-growing taxa such as many arbuscular mycorrhizal fungi (AMF), which cannot be easily cultured due to their dependency on host plants. The type of media used for fungal isolation may selectively promote fast-growing saprotrophic fungi while excluding oligotrophic or specialized species. Furthermore, competition between fungal species during cultivation can result in the overrepresentation of aggressive or dominant taxa, skewing community assessments. To address these limitations, integrated approaches combining culture-dependent and culture-independent methods (DNA metabarcoding and shotgun metagenomics) are increasingly being employed. For example, coupling traditional culturing with high-throughput sequencing can provide complementary insights into fungal diversity and function. Additionally, sampling strategies can also introduce biases due to variability in environmental factors, which exert a strong influence on fungal communities. Seasonal and spatial variability further complicate efforts to obtain representative samples. For instance, temporal changes in soil moisture can alter fungal activity and the community composition, while spatial heterogeneity in the soil structure can create microhabitats that favor different fungal species. To overcome this, samples were taken on the same day, exclusively from the rhizosphere of the roots with sufficient replication to capture the full extent of the variability. Moreover, the storage conditions prior to analysis, such as freezing or drying, may differentially affect the viability and detectability of certain fungi, which is why the sampling date was the same as the isolation day in our study. While significant progress has been made in soil fungal research, limitations and biases in isolation methods and the influence of environmental variability remain significant challenges. Continued methodological innovation, coupled with rigorous experimental design, will be essential for future comprehensive studies to advance our understanding of soil fungal ecology and its implications for ecosystem health. The utilization of soil fungal microflora as biofertilizers offers numerous advantages, including environmental friendliness, cost-effectiveness, and enhanced fertilizer utilization efficiency. However, in order to examine these possibilities, a deeper and more detailed analysis will be necessary.

## 4. Materials and Methods

The study was conducted via a long-term experiment during the 3 vegetation seasons of winter wheat, 2020/21–2022/23.

### 4.1. Soil Samples for Fungal Analyses

Winter wheat was sampled during the BBCH 55 [49] stage (half of inflorescence emerged/middle of heading) as some cropping systems may significantly differ at later growth stages (at the ripening phase). Usually, during this period, the temperate climate soil is sufficiently moist and the roots are highest in length and diameter [51]. The representative soil samples were taken from the surface of the rhizosphere layer (0–20 cm depth) using a soil probe. Soil samples were transferred in paper bags into the laboratory where fungal isolation was performed in the next 24 h.

### 4.2. Fungal Isolation and Identification

For fungal isolation, the particle-plating method was used because it yields higher numbers of taxa than the dilution plating method. Particle-plating method involved placing soil particles weighing 0.0001 g in a Petri dish with selective water agar (WA) media amended with streptomycin. A total of 35 soil pieces (5 pieces per Petri dish, in seven replicates) made of 0.0035 g of soil per sample were analyzed after seven days of incubation at room temperature (25–28 °C) in the dark. The emerging fungal colonies were first microscopically observed and, according to their sporulation, transferred to suitable media for further analysis of morphological characteristics (incubation conditions the same as previously described). Final identification was performed according to Leslie and Summerell [52] and Watanabe [53] identification keys. The total number of colonies of each species identified was recorded in samples. For further data analyses, the number of colonies of fungus species/0.0035 g soil/soil sample was converted to the abundance of soil fungus—number of colony forming units (CFUs) per g of soil sample.

### 4.3. Experimental Site

This study was performed in the southern part of the Pannonian Basin in Serbia. The experiments are situated at the Rimski Šančevi experimental station (45°19′ N, 19°50′ E) of the Institute of Field and Vegetable Crops in Novi Sad. The prevailing climate is continental, with an average annual precipitation of 643 mm and an average annual temperature of 11.8 °C. The climate is favorable for major crop production, including winter wheat, maize, soybean, and sunflower. During the experiment, the average annual temperature was 12.8 °C and the average precipitation was 625 mm, which was higher than the long-term yearly averages. The choice of crops in the crop rotation was set 70 years ago and consists of the most important arable crops from an agronomic and economic point of view, and they have not been changed since the beginning of the trial. Given the area and paramount importance of winter wheat in Pannonian plane, our study centered on this crop [20]. Since the beginning of the experiment, tillage has been carried out by plowing since almost 2018/2019, and reduced tillage has been introduced due to the obvious effects of climate change on plants. The soil is slightly Haplic Chernozem according to the IUSS Working Group WRB (2022) [54] developed on loess and loess-like sediments, with clay mineralogy dominated by illite (2:1 layer type). Soil in the investigated cropping system belongs to the clay loam texture class, with average values of sand 44.8–49.8%; silt 30.3–33.7%; and clay 19.8–22.7%. The selected soil analysis methods followed national regulations for soil sampling and most importantly, the same methods have been used since the beginning of the trial, which gives us the possibility of monitoring and comparing the obtained results.

The crop rotation experiment was established in 1946 as a single rotation in which each crop is grown every year on separate 90 × 30 m plots. For the purpose of our study the following cropping systems were assessed:

1. MOC—Winter wheat monoculture conservation tillage, established 2019/20, with 100 kg ha^−1^ N fertilization without crop residue incorporation, P and K based on soil analyses;
2. MOP—Winter wheat monoculture with plowing, established 1970/41, receiving 100 kg ha^−1^ N with fertilizers and with incorporation of the crops residue, P and K based on soil analyses;
3. N2P—Two-year rotation unfertilized, moldboard plowing, established 1946/47 (maize–winter wheat), incorporation of crop residues;
4. N3P Three-year rotation unfertilized, moldboard plowing, established 1946/47 (maize–soybean–winter wheat), incorporation of crop residues;
5. F2C—Two-year rotation fertilized, (maize–winter wheat), established 2019/20, conservation tillage with 100 kg ha^−1^ N, with the incorporation of the crop residue, P and K based on soil analyses;
6. F2P—Two-year rotation fertilized, (maize-winter wheat), established 1970/71, moldboard plowing, 100 kg ha^−1^ N, with the incorporation of the crops residue, P and K based on soil analyses;
7. F3C—Three-year rotation fertilized, (maize-soybean—winter wheat), established 2019/20, conservation tillage, with 100 kg ha^−1^ N, and incorporation of the crops residue, PK based on soil analyses;
8. F3P—Three-year rotation fertilized, (maize-soybean—winter wheat), established 1970/71 moldboard tillage, with 100 kg ha^−1^ N, and incorporation of the crop residue, PK based on soil analyses;
9. F3Ccc—Three-year rotation fertilized, (maize (winter oat)–soybean–winter wheat (field pea), with cover crop (winter wheat/field peas), established 2019/20, conservation tillage, with N fertilization of 100 kg ha^−1^, and incorporation of the crop residue, PK based on soil analyses;
10. F3Pcc—Three-year rotation fertilized, (maize (winter oat)–soybean–winter wheat (field pea)), with cover crop (winter wheat/field peas), established 1970/71, moldboard tillage, with fertilization of 100 kg ha^−1^ N, and incorporation of the crops residue, PK based on soil analyses.

Winter wheat sowing usually occurred between 20 and 30 October, with a seeding rate of 230–250 kg ha^−1^ with HORSH Pronto DC seeder. Weeds were sprayed at the beginning of April with Biathlon 5 g + Opus tim 1.2 L + Dash 0.2 L. During the observational period, the highest-yielding commercial wheat varieties grown were Zvezdana according to yield rankings in separate strip trials. On the plots where plowing is applied, the plant residues of the preceding crops are first chopped up and then plowed to a depth of 27 cm. In the spring, the furrows are closed in February, and then the pre-sowing preparation is carried out with the germinator machine. Nitrogen in wheat is applied in two doses at 100 kg per ha, with the first dose before plowing and the second in March.

Conservation tillage was based on the absence of tillage of the soil for wheat and the direct use of the working organs of the seeder and the sowing of seeds and the preparation of the soil, but, for both plowing and conservation tillage, the same seeder was used to maintain the same grain number per square meter.

### 4.4. Data Analysis

The diversity of different soil samples was analyzed by the Shannon–Wiener index (H′) and calculated according to the following formula:

$$H^{\prime }=-\sum_{i=1}^{n}p_{i}lnp_{i}$$

where p_i_ is the proportion of the number of colonies of the i-th species to the total number of colonies when i = 1, 2, 3, …n.

Furthermore, the relationship between soil physical and chemical properties and fungi was analyzed using the ordination method with canonical correspondence analysis (CCA). Canonical correspondence analysis (CCA) is a multivariate method convenient for clarification of the relationships between biological assemblages of species and their environment.

## 5. Conclusions

The results obtained revealed significant fungal diversity among the cropping systems studied, with predominant species, *Fusarium* spp., *Aspergillus* sp., and *Penicillium* sp., showing distinct preferences for different soil conditions. Conservation tillage plots were found to increase fungal diversity and promote beneficial phosphate-solubilizing fungi. Soils in the cropping systems with higher OM levels and total nitrogen were observed to be associated with increased fungal richness and diversity. This study could contribute to the customization of crop rotations and tillage systems in order to improve soil health and overall winter wheat productivity in temperate climatic conditions. Furthermore, the obtained results can provide guidelines for the transition to agroecological or regenerative agriculture strategies in the Chernozem soils of the Pannonian Basin.

## Figures and Tables

**Table 1:** Soil chemical properties of the different winter wheat-based systems in 0–30 cm (average of 2020–2023).

System	pH		CaCO_3_ %	OM %	Total N %	Al-P_2_O_5_ mg/100 g	Al-K_2_O mg/100 g
	KCl	H_2_O					
MOC	7.37 ± 0.05	8.29 ± 0.03	7.05 ± 1.40	2.57 ± 0.22	0.19 ± 0.01	32.73 ± 3.66	23.19 ± 2.98
MOP	7.14 ± 0.07	8.08 ± 0.08	1.19 ± 0.62	3.09 ± 0.49	0.22 ± 0.03	31.56 ± 2.71	35.84 ± 2.7
N2P	7.45 ± 0.05	8.38 ± 0.03	9.10 ± 1.40	2.10 ± 0.22	0.17 ± 0.01	5.98 ± 3.66	15.86 ± 2.98
N3P	7.39 ± 0.05	8.30 ± 0.03	6.63 ± 1.40	2.40 ± 0.22	0.18 ± 0.01	5.77 ± 3.66	17.06 ± 2.98
F2C	7.38 ± 0.03	8.22 ± 0.08	5.90 ± 0.93	2.36 ± 0.08	0.17 ± 0.01	71.23 ± 11.06	29.50 ± 3.30
F2P	7.30 ± 0.05	8.20 ± 0.09	3.20 ± 1.77	2.53 ± 0.14	0.19 ± 0.02	48.15 ± 22.90	33.79 ± 4.98
F3C	7.44 ± 0.07	8.30 ± 0.12	7.94 ± 2.84	2.41 ± 0.24	0.18 ± 0.01	101.65 ± 30.67	42.89 ± 12.17
F3P	7.39 ± 0.04	8.32 ± 0.09	6.86 ± 3.78	2.38 ± 0.19	0.17 ± 0.02	91.17 ± 19.35	41.00 ± 6.93
F3Ccc	7.38 ± 0.04	8.29 ± 0.04	5.68 ± 0.97	2.88 ± 0.14	0.20 ± 0.02	118.00 ± 25.68	52.44 ± 9.88
F3Pcc	7.32 ± 0.10	8.20 ± 0.10	5.50 ± 3.04	2.87 ± 0.21	0.21 ± 0.01	135.50 ± 17.41	68.00 ± 17.22

Legend: MOC: WW monoculture CT; MOP: WW monoculture PT; N2P: unfertilized WW PT; N3P: unfertilized WW PT; F2C: fertilized 2-year WW CT; F2P: fertilized 2-year WW PT; F3C: fertilized 3-year WW CT; F3P: fertilized 3-year WW PT; F3Ccc: fertilized 3-year WW cover crop CT; F3Pcc: fertilized 3-year WW cover crop PT.

**Table 2:** The abundance of soil fungi (CFU/g dry weight soil/soil sample × 10^3^) identified in ten soil samples during the three-year period/2020–2023 period.

2019–2023 Abundance (CFU/g × 10^3^)											
No	Species	MOC	MOP	N2P	N3P	F2C	F2P	F3C	F3P	F3Ccc	F3Pcc
1	*Acremonium* sp.	1.67	1.83	0.5	0.67		0.67	1.0	0.5	1.33	1
2	*Alternaria* sp.	2.67	0.17		0.5	0.5	1.83	2	2.17	2.3	2.5
3	*Aspergillus* sp.	1.17		0.83	1	0.67	0.5	1	0.83	0.5	0.17
4	*Bipolaris* sp.	0.17									0.83
5	*Chaetomium* sp.			0.17						0.3	
6	*Cunninghamella* sp.	0.17			0.17			0.17			
7	*Epicoccum* sp.						0.33	0.17			
8	*F. graminearum*				0.17						
9	*F. oxysporum*	3.33	4.83	3.17	4.0	3.2	3.83	4.33	2.17	4.83	7.0
10	*F. proliferatum*		0.17	0.17		0.17	0.67				0.2
11	*F. pseudograminearum*		0.17								
12	*F. semitectum*										0.2
13	*F. solani*	1.17	2.83	2.3	0.67	2		1.17	1.33	1.5	2.5
14	*F. verticillioides*										0.17
15	*Fusarium* sp.	0.17							0.17		
16	*Gliocladium* sp.			1.8	2.5	4.3	3.0	3.3	2.83	3.3	1.0
17	*Hyalodendron* sp.	0.5	0.5		0.17	0.5	0.67	0.17	0.3	0.3	0.5
18	*Macrophomina phaseolina*	2	1	4.83	5	3.3	2.33	1	1.7	2.8	0.5
19	*Micelia sterilia*	0.33	0.17		0.3	0.17		0.7	0.83		0.3
20	*Mortierella* sp.	4.17	2.83	2.5	3.8	3.67	3.7	1.5	2.7	3	2.5
21	*Mucor* sp.		0.17			0.17				0.33	
22	*Paecilomyces* sp.	0.5		0.33	0.5		0.17	0.7	0.17	0.50	0.17
23	*Penicillium* sp.	2.3	2.67	3.5	1.33	1.7	0.5	2.5	1.33	1.00	0.67
24	*Pythium echinulatum*	2.17	0.83	2	2.5	1.67	2.17	2.33	1	2.33	2
25	*Pythium* sp.	1.67	1.67	1.3	2.0	1.0		1.5	2.8	2.00	1
26	*Ramichloridium* sp.		0.17		0.5	1.5	0.83	0.33		0.17	1.3
27	*Rhizopus* sp.	0.5	1.5		0.17	0.67	0.33	0.17	0.17	0.17	0.17
28	*Staphylotrichum* sp.				0.17					0.5	
29	*Thielaviopsis* sp.		0.17		0.17	0.17		0.67	0.17	0.33	
30	*Trichoderma* sp.			0.67	1.33	1.5	1	0.8	0.83	0.33	0.67
31	*Trichothecium* sp.				0.17	0.17					
32	*Ulocladium* sp.								0.5		
Total average CFU/g × 10^3^ per sample		24.69	21.68	24.16	27.85	27.03	22.5	25.5	22.5	27.97	25.35
Average No of species per sample		17	17	14	22	19	16	20	18	20	21

Legend: MOC: WW monoculture CT; MOP: WW monoculture PT; N2P: unfertilized WW PT; N3P: unfertilized WW PT; F2C: fertilized 2-year WW CT; F2P: fertilized 2-year WW PT; F3C: fertilized 3-year WW CT; F3P: fertilized 3-year WW PT; F3Ccc: fertilized 3-year WW cover crop CT; F3Pcc: fertilized 3-year WW cover crop PT.

**Table 3:** Average number of species for three-year period, average abundance, and Shannon–Wiener’s diversity index of soil fungi in examined WW cropping systems.

Soil Sample	Average No of Species	Average Abundance (CFU/g × 10^3^ of Soil)	Shannon–Wiener’s Diversity Index
MOC	17	24.69	1.90
MOP	17	21.68	2.09
N2P	14	24.16	2.08
N3P	22	27.85	2.25
F2C	19	27.03	2.38
F2P	16	22.50	2.04
F3C	20	25.51	2.17
F3P	18	22.00	2.15
F3Ccc	20	27.97	2.11
F3Pcc	21	25.35	1.99

## Data Availability

The data are contained within this manuscript. All data, tables, and figures in this manuscript are original.
